# Non-Invasive Diagnosis of Early Breast Cancer: Current and Emerging Liquid Biopsy Biomarkers

**DOI:** 10.3390/cancers18142344

**Published:** 2026-07-20

**Authors:** Amalia Kotsifaki, Charikleia-Rafaela Masoura, Georgia Limogianni, Georgia Kalouda, Martha Stathaki, Athanasios Armakolas

**Affiliations:** 1 Physiology Laboratory, Medical School, National and Kapodistrian University of Athens, 11527 Athens, Greece; amkotsifaki@med.uoa.gr (A.K.); chmasoura@biol.uoa.gr (C.-R.M.); georgialimo@biol.uoa.gr (G.L.); gkalouda@med.uoa.gr (G.K.); 2 Department of Surgery, Elena Venizelou Hospital, 11521 Athens, Greece; stathakimg@yahoo.gr

**Keywords:** breast cancer, liquid biopsy, early detection, tumor microenvironment, circulating tumor DNA (ctDNA), circulating tumor cells (CTCs), extracellular vesicles (EVs), circulating biomarkers, tumor heterogeneity, multi-omics

## Abstract

**Background/Objectives:** Breast cancer (BC) remains the most frequently diagnosed malignancy among women worldwide, and patient outcome is strongly influenced by disease stage at diagnosis. Although imaging-based screening has improved early detection, its performance may be reduced in dense breast tissue and is associated with false-positive findings. In addition, tissue biopsy is invasive and unsuitable for longitudinal disease monitoring. Liquid biopsy (LB) has emerged as a minimally invasive approach for detecting tumor-derived material in peripheral blood. However, early-stage tumors typically exhibit low tumor burden and limited biomarker shedding, generating weak systemic signals that challenge reliable detection. This review examines current and emerging LB biomarkers for early BC detection. **Methods:** A comprehensive review of recent literature was conducted focusing on circulating tumor cells (CTCs), circulating tumor DNA (ctDNA), extracellular vesicles (EVs), circulating RNAs, proteins, and other blood-based biomarkers associated with early BC. Studies addressing biomarker biology, detection technologies, clinical applications, and methodological limitations were critically evaluated. **Results:** ctDNA, CTCs, EVs, circulating RNAs, proteins, and additional blood-based biomarkers capture distinct aspects of tumor biology and disease evolution. ctDNA enables the analysis of tumor-specific mutations, methylation patterns, and fragmentation profiles, whereas CTCs provide direct cellular and phenotypic information despite their rarity and marked epithelial–mesenchymal plasticity. EVs offer increased molecular stability and actively participate in tumor progression, immune modulation, and metastatic niche formation. Nevertheless, low biomarker abundance, biological heterogeneity, technical variability, and background biological noise continue to limit analytical performance, particularly in early-stage disease. Current evidence further suggests that no single biomarker consistently provides sufficient sensitivity and specificity for reliable early BC detection. **Conclusions:** LB represents a promising strategy for non-invasive early BC detection. Future clinical implementation will likely depend on integrated multi-analyte approaches that combine complementary genomic, transcriptomic, proteomic, and cellular information, supported by multi-omics technologies and artificial intelligence-based analytical frameworks.

## Simple Summary

Breast cancer (BC) is one of the most common cancers worldwide, and early diagnosis is essential for improving patient outcomes. Current screening methods, such as mammography, have significantly improved early detection but still have limitations, particularly in women with dense breast tissue. In addition, traditional tissue biopsies are invasive and are not well suited for longitudinal disease monitoring. Liquid biopsy (LB) is a promising blood-based approach that detects material released by tumors into the bloodstream, offering a minimally invasive alternative for cancer detection and monitoring. This review summarizes the most important LB biomarkers being investigated for the early detection of BC, including circulating tumor DNA (ctDNA), circulating tumor cells (CTCs), extracellular vesicles (EVs), and other blood-based markers. Major biological and technical barriers continue to restrict clinical translation, while emerging analytical and technological advances may improve the sensitivity, specificity, and overall robustness of early BC detection.

## 1. Introduction

Breast cancer (BC) remains the most frequently diagnosed malignancy among women worldwide and one of the leading causes of cancer-related mortality [1]. In 2022, approximately 2.3 million new cases were reported globally, and current projections suggest that the incidence will continue to rise substantially over the coming decades [2]. Importantly, clinical outcome is strongly determined by the stage at diagnosis. While patients with localized disease achieve excellent long-term survival, prognosis deteriorates considerably following regional or distant dissemination [3]. Consequently, detecting BC at its earliest clinically actionable stage remains one of the most effective strategies for reducing disease-associated mortality and improving patient outcomes [4].

Population-based mammographic screening has significantly contributed to earlier diagnosis and declining mortality rates in many countries. However, current imaging modalities remain imperfect, particularly in the setting of early disease detection [5]. Mammographic sensitivity is reduced in women with dense breast tissue, small lesions may escape radiological detection, and false-positive findings frequently lead to unnecessary investigations and patient anxiety [6,7]. Furthermore, concerns regarding overdiagnosis continue to fuel debate regarding the optimal implementation of screening programs. Collectively, these limitations highlight the need for complementary diagnostic approaches that detect tumor-associated alterations before they become visible through conventional imaging [8].

Tissue biopsy remains the gold standard for BC diagnosis and molecular characterization, providing essential information regarding tumor subtype, receptor status, grade, and therapeutic targets [9]. Nevertheless, this approach is invasive and provides information from only a single time point of disease evolution. BC exhibits substantial spatial and temporal heterogeneity, driven by the continuous evolution of genetically and phenotypically distinct cellular populations [10]. Consequently, a single tissue specimen may fail to capture the full molecular complexity of the tumor, particularly as selective pressures imposed by the microenvironment and therapeutic interventions reshape tumor composition over time. In addition, repeated tissue sampling is often impractical, limiting its utility for longitudinal disease monitoring [11].

Because of these limitations, there has been growing interest in minimally invasive approaches that enable longitudinal molecular monitoring of disease evolution. Among them, liquid biopsy (LB) has emerged as a non-invasive strategy that analyzes tumor-derived material released into biological fluids, most commonly peripheral blood, to provide insight into the molecular characteristics of cancer [12,13]. A wide range of circulating biomarkers can be detected through LB, including circulating tumor cells (CTCs), circulating tumor DNA (ctDNA), extracellular vesicles (EVs), circulating RNAs, proteins, metabolites, and other analytes collectively provide insights into the biological state of the tumor [14,15]. Unlike conventional tissue biopsy, which reflects a single site and time point, LB enables repeated sampling over the course of disease, thereby facilitating longitudinal assessment of tumor-associated changes and disease evolution [14].

The LB landscape encompasses a diverse spectrum of biomarkers that differ considerably in their biological origin, abundance, stability, and clinical relevance [16]. CTCs provide direct evidence of tumor cell dissemination, ctDNA enables the detection of tumor-specific genetic and epigenetic alterations, and EVs serve as active mediators of intercellular communication through the transfer of proteins, lipids, and nucleic acids [17,18]. Other circulating analytes, including non-coding RNAs, proteins, metabolites, and immune-derived biomarkers, may capture additional dimensions of tumor biology as well as host responses to malignant transformation [19]. Together, these biomarkers provide complementary information and support the development of multi-analyte approaches for BC detection [18]. Increasingly, these approaches are being combined with multi-omic profiling strategies to capture the complex biological interactions that underpin early tumor development and progression [15].

Beyond its minimally invasive nature, LB offers a unique opportunity to address one of the central challenges in BC management: tumor heterogeneity [9,14]. Unlike localized tissue sampling, circulating biomarkers may originate from multiple tumor regions and cellular subpopulations, potentially providing a broader representation of disease biology [20]. This capability has generated considerable interest in the application of LB across a wide range of clinical settings, including early detection, disease monitoring, minimal residual disease assessment, prognostication, and therapeutic response evaluation [14].

Despite remarkable technological progress, the application of LB to early BC detection remains particularly challenging. Although technological limitations remain important, the main obstacle to early BC detection appears to be biological in nature [12,21]. Early-stage tumors are characterized by limited tumor mass, restricted vascular interaction, and comparatively low rates of biomarker release into the circulation [22]. As a result, tumor-derived analytes are often present at extremely low concentrations, frequently approaching the limits of analytical detection. Thus, the difficulty of identifying early disease reflects not only technological limitations, but also the inherently weak biological signals generated by incipient tumors [23].

Early-stage BCs may therefore be regarded as biologically silent systems that leave only a minimal systemic footprint [24]. Prior to extensive angiogenesis, tissue remodeling, and metastatic dissemination, communication between the developing tumor and the systemic circulation remains relatively limited [9,15]. As a result, tumor-derived biomarkers are frequently present at very low concentrations and are difficult to distinguish from normal circulating components. Under these conditions, distinguishing genuine tumor-associated signals from physiological background noise becomes exceptionally difficult [25]. This unfavorable signal-to-noise ratio represents one of the principal barriers to the successful implementation of LB-based screening strategies and may partly explain the variable performance reported across different biomarker classes [23].

Recent advances in high-sensitivity sequencing technologies, digital nucleic acid analysis, single-cell platforms, and multi-omic profiling have substantially expanded the ability to detect rare circulating biomarkers [15]. These developments have accelerated biomarker discovery and broadened the scope of LB research in BC [9]. Nevertheless, analytical sensitivity alone is unlikely to overcome the challenges associated with early disease detection. Equally important is a deeper understanding of the biological processes that govern biomarker generation, release, transport, and persistence within the circulation [25,26].

Importantly, circulating biomarkers should not be considered passive by-products of tumor growth. Their abundance and composition are shaped by complex interactions among tumor cells, stromal populations, vascular networks, immune cells, and the broader tumor microenvironment (TME) [14,27,28]. Processes such as cell death, active secretion, intravasation, early dissemination, and tumor dormancy collectively influence the quantity and characteristics of tumor-derived material entering the circulation [9,29]. Consequently, the detectability of circulating biomarkers is intrinsically linked to tumor biology, reflecting the complex mechanisms that govern tumor shedding and systemic signal generation.

In this context, the present review examines current and emerging LB biomarkers for the non-invasive detection of early BC. Particular emphasis is placed on the biological mechanisms governing tumor shedding, the potential of circulating biomarkers to capture early tumor-associated signals, and the technological and clinical barriers that continue to hinder their translation into routine clinical practice. This study highlights current evidence regarding liquid biopsy biomarkers in early BC and discusses the biological and clinical challenges that may influence their future application.

## 2. Biology of Early Tumor Shedding

The origin of circulating biomarkers in early BC is directly linked to the biological processes driving the release of tumor-derived material into the circulation [30]. The primary source of cell-free DNA (cfDNA) is apoptosis, a highly controlled and programmed form of cell death. This is evidenced by the distinct size spread of cfDNA fragments in the blood, which typically centers around 167 base pairs, a characteristic molecular pattern pointing directly to an apoptotic origin [31].

In contrast, as early tumors grow, some cells undergo necrosis. Unlike apoptosis, the disorganized form of cell death involves an unregulated and random breakdown of the cell’s genetic material, releasing much larger and highly variable DNA fragments into the circulation [32]. Because necrosis lacks enzymatic control, the cell membrane simply breaks in, spilling massive DNA strands, directly into the surrounding tissue and bloodstream [33]. Importantly, elevated levels of circulating genetic material may reflect not only enhanced release from dying tumor cells but also inefficient clearance of ctDNA by phagocytic cells [14]. Furthermore, biomarker shedding is also driven by active biological processes that contribute to the release of tumor-derived material into circulation. Active secretion is also a major contributor. Beyond freely circulating cfDNA, genetic material can be released into the circulation linked to or encapsuled within EVs released by cells [31]. These vesicles provide a crucial bilayer that protects the encapsuled biological cargo from rapid enzymatic degradation, thereby increasing their stability and circulating half-life of these molecular markers within the bloodstream [33,34].

The entry of tumor-derived material into the circulation further depends on the ability of tumors to access the vascular network. Intravasation serves as the definitive biological getaway that regulates the active and passive translocation of both viable malignant cells and tumor-derived biomarkers from the primary tumor into the systemic circulation [35,36]. The transition of these markers from the local tissue into the bloodstream is heavily dependent on the local vasculature [36]. In the early stages of BC, the growing tumor requires an increased blood supply and initiates angiogenesis, the formation of new blood vessels. However, these blood vessels are not structurally normal because unlike healthy vasculature, they are poorly organized and lack tight connections between their endothelial cells [37]. As a result, the local microvasculature becomes highly permeable, making a leaky network. The physical barrier facilitates the escape of cfDNA, exosomes and intact tumor cells through the endothelial cells straight into the bloodstream [36]. Moreover, these intact tumor cells must undergo some specific morphological changes to actively get into these blood vessels and spread as CTCs [14]. A major biological challenge in diagnosing early BC is the fact of early dissemination. In opposition to the basic progression model, which prescribes that tumor spread happens only in advanced stages, accumulating evidence indicates a parallel progression model [38]. In this condition, BC cells can actively disconnect from the primary area and disseminate into circulation, before the primary tumor becomes clinically or radiologically detectable. This early cellular spread creates the state of minimal residual disease (MRD), where micrometastases secretly seed distant organs but remain temporarily inactive [39].

Once these early circulating cells successfully extravasate into distant organs, they transition into disseminated tumor cells (DTCs) and reside in a prolonged dormant state [35]. The biological consequences of this early cellular spread are critical for long-term clinical outcomes. These hidden DTCs form a persistent cellular reservoir that can evade immune surveillance and remain clinically silent for decades within specialized niches like the bone marrow [40]. Crucially, these early-disseminating cells frequently share a close clonal relationship with subsequent lethal metastases, directly implicating early DTCs as the true evolutionary precursors of disease relapse [35].

**Figure 1:**
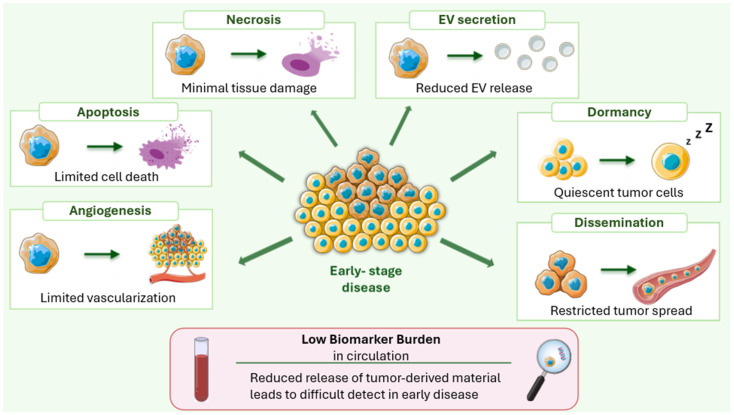
“Biological determinants of low biomarker shedding in early-stage breast cancer”. Schematic representation of the biological mechanisms contributing to the limited release of tumor-derived material into the circulation during early-stage breast cancer (BC). Small tumor burden, restricted vascularization, limited apoptosis and necrosis, reduced extracellular vesicles (EVs) secretion, cellular dormancy, and constrained tumor dissemination collectively decrease the systemic shedding of tumor-associated biomarkers. As a result, circulating tumor-derived signals, including ctDNA, CTCs, EVs, and other blood-based biomarkers, remain scarce, creating substantial challenges for the sensitive detection of early-stage disease through liquid biopsy (LB) approaches.

Following early dissemination, the detached tumor cells that survive in distant organs often enter a extended state of dormancy [38]. During this phase, these cells do not actively multiply. This balance between proliferation and apoptosis allows them to evade conventional cancer therapies, that typically target rapidly separating cells and keep them clinically silent for years [35,39]. In BC, this latency is extremely prevalent, as approximately 20% to 45% of patients eventually experience metastatic relapses, years after their treatment [41]. The localized biological dormancy directly underlies why early tumors fail to generate detectable systemic signals. Because DTCs remain in non-proliferative state and maintain a strict equilibrium between cell division and low-level apoptosis, they do not expand their biomass [40]. As a result, the active release of cellular breakdown products or genomic material into the circulation is minimized (Figure 1). During these early stages of disease progression, the total systemic biomarker burden remain restricted, preventing the generation of signals robust enough to overcome physiological clearance mechanisms [35,42].

The restriction of biomarker shedding is fundamentally regulated by the TME and specific tumor-immune interactions [42].Within anatomical reservoirs like the bone marrow perivascular niche, DTCs depend on microenvironmental signals from endothelial and stromal cells to maintain their inactive state [41]. At the same time, active immune surveillance plays a decisive role in controlling this latent population [42]. This tight bidirectional crosstalk between the dormant cells and the immune microenvironment suppresses active cell turnover and tissue remodeling [35]. Thus, although biological shedding begins early during tumor development, the limited tumor burden, restricted vascular access, dormancy of disseminated cells, and efficient physiological clearance mechanisms collectively contribute to the low abundance of circulating biomarkers in early-stage BC (Figure 1).

## 3. Circulating Tumor Cells (CTCs)

### 3.1. Biological Role and Metastatic Potential of CTCs

CTCs are cancer cells that detach from the primary tumor and circulate throughout the body via blood or lymphatic vessels [43,44]. Evidence indicates that thousands of cells are released from tumors into the circulation every day, most of them undergoing immediate apoptosis, with a survival time of about 1–2.5 h [45]. CTCs present one of the main causes of metastasis, as they are able to adhere to endothelial membranes of distant organs, after undergoing epithelial–mesenchymal transition (EMT), and there, form secondary tumors [46].

Traditionally, EMT is known to drive metastasis by downregulating epithelial markers and upregulating mesenchymal markers [47]. This loss of cell adhesion allows cancer cells to detach from the primary tumor and enter the bloodstream, a process further aided by the high expression of proteases and integrins [14]. Moreover, EMT induces stem cell characteristics, enabling asymmetric division where one cell retains self-renewal properties (cancer stem cells, CSCs) while the other forms the tumor mass [48]. However, contrary to the long-standing belief that complete EMT drives metastasis, emerging evidence shows that metastases are rarely initiated by exclusively mesenchymal CTCs. Instead, CTCs exhibiting a hybrid/intermediate epithelial/mesenchymal (E/M) phenotype possess enhanced phenotypic plasticity and CSC-like properties, rendering them uniquely capable of successfully colonizing distant metastatic sites [49,50].

The metastatic potential and survival of CTCs depend heavily on their EMT phenotype. While epithelial CTCs struggle to extravasate, due to the expression of epithelial markers, fully mesenchymal CTCs are highly migratory but vulnerable to fluid shear stress and lack the proliferative capacity to colonize secondary sites [51]. Crucially, hybrid epithelial/mesenchymal (E/M) CTCs exhibit the highest metastatic efficiency, as they frequently travel as multi-cellular clusters, resulting in a dramatically higher survival compared to single cells [52,53]. Thus, the phenotypic spectrum of CTCs directly dictates their fate in the bloodstream and their success in establishing metastases.

Translating these biological insights into clinical practice remains a great challenge, particularly in early-stage BC, where CTCs are found in extremely low concentrations. Unlike metastatic settings, CTCs in early BC are exceedingly rare, with as few as one single cell potentially present amidst millions of leukocytes and billions of erythrocytes per milliliter of blood [39]. This extreme scarcity, combined with the down-regulation of classical epithelial markers during partial EMT, demands the development of new-generation isolation technologies with significant sensitivity to avoid false-negative results and CTC misclassification [54].

CTC counts have been recognized as independent diagnostic and prognostic indicators in several malignancies, including BC [55,56]. In particular, in metastatic BC, the detection of five or more CTCs per 7.5 mL of blood has been consistently associated with shorter progression-free survival, reduced overall survival, and increased mortality risk [57,58,59]. Given their strong clinical relevance, CTCs have attracted considerable scientific interest since their first description by the Australian pathologist Thomas Ashworth in 1869 [60,61]. Since then, extensive research efforts have focused on developing reliable methods for their detection, isolation, and molecular characterization. However, despite their established clinical significance, the accurate identification of CTCs remains technically challenging because of their extreme rarity in the circulation, typically ranging from 1 to 10 cells per mL of blood [9,62]. Consequently, a variety of enrichment and isolation strategies have been developed to improve their recovery and enable downstream phenotypic and molecular analyses.

### 3.2. CTC Isolation and Detection Technologies

With a diameter typically ranging from 16 to 20 μm, CTCs are significantly larger than red blood cells (~8 μm) and white blood cells (~8–14 μm). This distinct size differential is widely exploited to isolate them from peripheral blood [44].Regarding the filtration devices for CTC isolation, they vary significantly in pore size, material, and architecture. Following isolation, captured cells can be either visualized on glass slides or processed for downstream molecular analysis [44]. However, these size-based methods often suffer from limited purity compared to functional assays, as they fail to fully account for the inherent heterogeneity of CTCs [60].

Given that most cancers arise from epithelial tissues, the initial strategy for CTC isolation relied on targeting epithelial antigens. Among these, the epithelial cell adhesion molecule (EpCAM) quickly became the gold standard for CTC identification in the bloodstream [63]. EpCAM is a transmembrane glycoprotein expressed in both healthy epithelial tissues and epithelial cancer cells [64]. It is essential for cancer cell adhesion, proliferation, migration, and EMT. Its positive correlation with Ki67 makes it a valuable prognostic marker [65]. While highly expressed in various carcinomas, including breast, colon, and prostate, its presence is limited in melanomas, lymphomas, and sarcomas [64].

The CellSearch system is the only FDA-approved platform for EpCAM-based CTC enrichment, employing anti-EpCAM-coated magnetic nanoparticles for positive selection [49]. While highly effective in metastatic settings, its reliance on epithelial markers limits its sensitivity for detecting CTCs undergoing EMT. Other systems, like the magnetic-activated cell sorting system (MACS) enable the enrichment of CTCs by labeling them with antibody-coated magnetic MicroBeads, achieving sensitivity as high as 1 cell per 107 blood cells [62]. Although EpCAM-based isolation is efficient for many epithelial cancers, its reliability is limited by the phenotypic plasticity of CTCs. During EMT, CTCs frequently downregulate epithelial markers like EpCAM and E-cadherin [66]. Partial EMT and hybrid (E/M) phenotypes further hinder the classification of CTCs, making EpCAM an unreliable marker for their isolation [67].Taken together, these drawbacks emphasize the limitations of EpCAM for CTC capture, underscoring the necessity for alternative approaches. To address this limitations, other methods have integrated alternative tumor-specific markers, including EGFR, HER2, folic acid receptors (FRs) and recombinant VAR2CSA (rVAR2) [68,69,70].

Functional assays exploit the biological traits of viable CTCs to address intratumoral heterogeneity, though they frequently struggle with suboptimal purity compared to other enrichment techniques. A prominent example is the Epithelial Immunospot (EPISPOT) assay, an adaptation of the enzyme-linked immunospot technology, designed to identify viable CTCs via the highly sensitive immunocapture of secreted, shed, or released proteins during brief ex vivo incubation [71]. Similarly, the collagen adhesion matrix (CAM) assay serves as a functional separation tool to isolate highly aggressive CTCs for molecular characterization [44,72].

Microfluidics has emerged as a novel effective technology that enables fluid manipulation at the microscale. It allows for continuous sample processing that minimizes target cell loss and can capture CTCs through different methods, including epithelial antigen targeting and the exploitation of cellular physical or electrical properties [73]. CTC-chip is a microfluidic that employs antibody-coated microposts to isolate EpCAM-positive CTCs [44]. Over the past decade and a half, many similar platforms have been developed [60,74,75,76,77]. Although microdevices present a highly promising approach for CTC isolation and enrichment, they are still hindered by significant challenges, particularly regarding suboptimal sample purity and the aforementioned limitations associated with the use of EpCAM antibodies.

**Figure 2:**
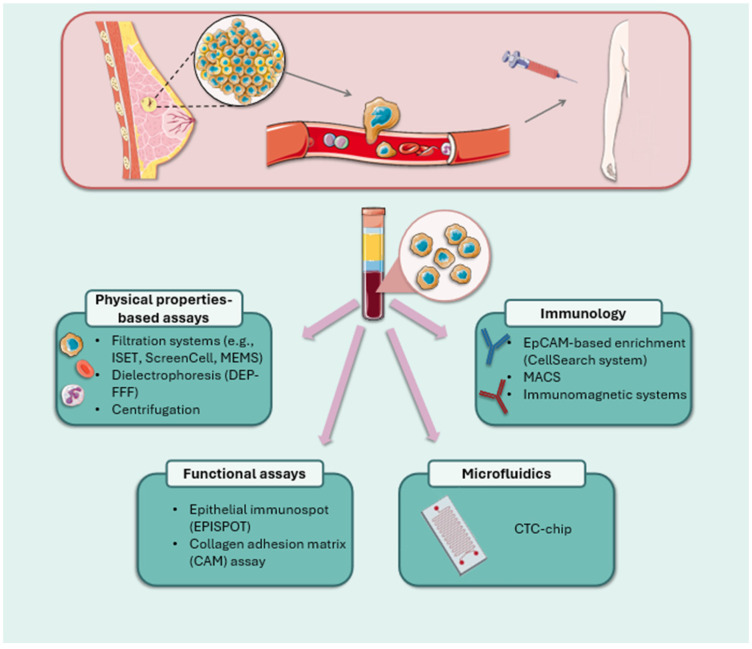
“Overview of CTC isolation methodologies”. Dissemination of circulating tumor cells (CTCs) from the primary tumor into the bloodstream and subsequent capture via liquid biopsy (LB). Enrichment strategies are classified into four key categories: Physical properties (e.g., filtration, dielectrophoresis, centrifugation), Immunological capture (e.g., EpCAM-based enrichment, MACS), Functional assays (e.g., EPISPOT, CAM assay), and Microfluidic platforms (e.g., CTC-chip).

Following CTC enrichment and isolation, a range of analytical approaches are employed for their detection, enumeration, and molecular characterization [78]. Immunocytochemistry (ICC) and flow cytometry are cornerstone visual and quantitative techniques for CTC analysis. ICC utilizes fluorescent monoclonal antibodies with automated imaging for CTC visualization, whereas flow cytometry allows rapid quantification of surface and intracellular antigens, providing high-throughput profiling [79,80]. In BC management, these methods are critical for assessing tumor heterogeneity and molecular profiling [81]. Moreover, flow cytometry has been instrumental in characterizing BC-derived CTC clusters, which, despite their rarity, are strongly linked to increased metastatic efficiency and poor clinical outcomes [82]. Modern optical detection predominantly relies on image-based cytometry platforms, most notably automated digital microscopy (ADM) and fiber-optic array scanning technology (FAST), which execute automated visual analysis of ICC labeled tumor populations [83]. To circumvent these limitations, FAST was introduced as a high-velocity alternative, offering equivalent diagnostic sensitivity and superior specificity while accelerating the scanning rate [83]. The major approaches currently used for CTC enrichment, isolation, and downstream characterization are summarized in Figure 2.

In conclusion, CTCs serve as a potent, non-invasive biomarker for real-time monitoring of tumor evolution and metastatic progression. While they offer a unique window into BC biology, their clinical translation, especially in early-stage disease, remains hindered by extreme cell rarity, phenotypic plasticity, and methodological heterogeneity. Bridging this gap requires the integration of label-independent enrichment technologies with comprehensive, multi-marker detection strategies. Such advancements are crucial to capturing the full spectrum of CTC plasticity and standardizing their application in clinical practice.

## 4. Circulating Tumor DNA (ctDNA)

CtDNA is considered a subset of cfDNA that consists of single- or double-stranded DNA fragments released from tumor cells, either in primary, or in metastatic sites [84]. These fragments can be detected in peripheral blood, including in some patients with early-stage disease, making ctDNA a promising biomarker for disease monitoring, prognostic assessment, and treatment guidance. Moreover, ctDNA contributes in the assessment of post-surgical MRD [85]. Unlike traditional screening methods, ctDNA provides a dynamic, real-time reflection of the disease, offering more precise guidance for BC diagnosis and treatment.

Similarly to cfDNA, ctDNA enters the bloodstream through two main processes: passive release from dying or injured cells (such as those undergoing apoptosis, pyroptosis, necrosis or autophagy) and active secretion by living cells [86,87]. In cancer patients, the total cfDNA pool is a mixture of DNA from healthy cells and malignant cells from the TME [87]. ctDNA specifically refers to tumor-derived DNA released from malignant cells and, to a lesser extent, other cellular components of the TME [86,88]. Additionally, CTCs and exosomes act as key sources of ctDNA, as they are shed by the primary tumor or metastatic sites into the bloodstream [43,89,90].

**Figure 3:**
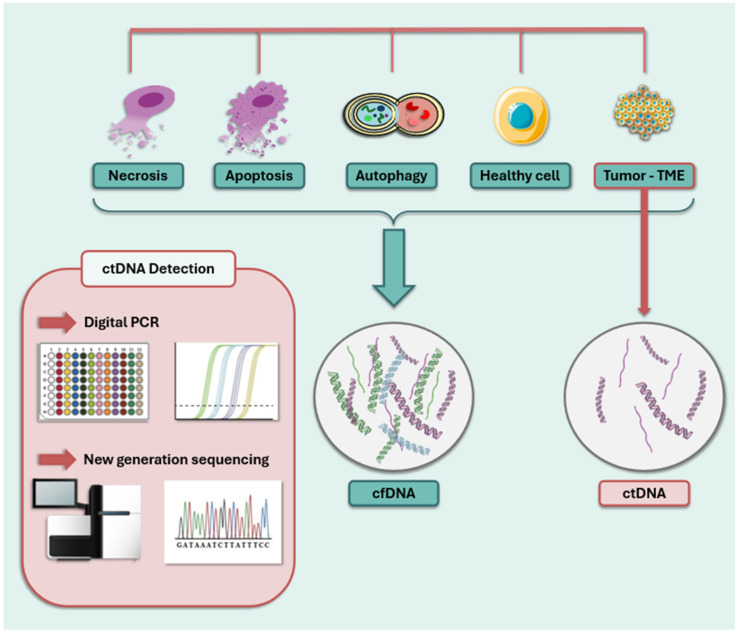
“Biological origin of cfDNA and ctDNA and current approaches for ctDNA detection”. Release of cfDNA via cellular processes (apoptosis, necrosis, autophagy) and distinction between healthy-derived cfDNA and tumor-derived ctDNA from the tumor microenvironment (TME). Analytical approaches, including Digital PCR and Next-Generation Sequencing (NGS), are then employed to identify tumor-specific genomic alterations.

Although ctDNA is part of the total cfDNA pool, it is unique because it is found only in cancer patients. Unlike normal cfDNA, that primarily originates from the apoptosis of hematopoietic cells, ctDNA carries tumor-specific markers, such as genetic mutations and abnormal methylation, which help identify malignant cells [91,92]. There are also structural differences between these DNA fragments, especially regarding their size. cfDNA from apoptotic cells often appears in lengths of about 166 bp (the size of a nucleosome), or longer, while tumor-derived ctDNA is frequently highly fragmented, often measuring less than 100 bp [85]. Fragmentation patterns may also be used in order to differentiate ctDNA from cfDNA [93,94,95]. In terms of quantity, studies on various cancers, including BC, show that cfDNA levels in patients is 3 to 30 times higher than in healthy individuals, with the highest levels found in metastatic disease [85]. ctDNA usually makes up only a small fraction of the total cfDNA, despite the fact that tumor growth and cell death lead to the release of a great amount of DNA [39]. The amount of ctDNA in the blood, though, is directly related to tumor burden. This fraction varies significantly depending on the stage of the disease: it typically represents less than 1% of total cfDNA in early-stage cancers, but can rise to over 90% in advanced, late-stage disease [96]. All the above suggest that ctDNA detection greatly contributes to the assessment of disease progression and prognosis. The biological origins of cfDNA and ctDNA, together with the principal analytical approaches currently used for ctDNA detection, are summarized in Figure 3.

Nowadays, ctDNA detection is mainly performed with precise and diverse technologies, such as digital PCR (dPCR) and next-generation sequencing (NGS) among others [97]. dPCR represents a transformative advancement in molecular diagnostics, facilitating the absolute quantification of nucleic acids through single-molecule amplification [98]. The technology is primarily categorized into Droplet Digital PCR (ddPCR) and Chip-based Digital PCR (cdPCR), with the former currently serving as the industry standard for LB applications [99,100]. The ‘digital’ nomenclature arises from the system’s binary readout, where the reaction mixture is partitioned into thousands of isolated micro-reactors; each unit serves as an independent assay, providing a ‘positive’ or ‘negative’ signal that enables absolute quantification [101]. Well known commonly mutated genes in BC, such as PIK3CA and ESR1 have been targeted by dPCR, offering valuable information regarding patient survival and treatment outcomes [102].

Recent advancements in NGS have expanded the landscape of non-invasive molecular profiling, enabling the comprehensive detection of genomic alterations without the requirement for prior tumor-tissue knowledge [96]. By facilitating the simultaneous interrogation of multiple patient-specific genomic aberrations, these NGS-based frameworks provide a high-resolution view of tumor evolution [103]. Such depth of analysis is particularly vital for the clinical management of heterogeneous malignancies characterized by profound genomic instability, where traditional diagnostic approaches may fail to capture the full spectrum of clonal complexity.

Beyond traditional mutation detection, DNA methylation and cfDNA fragmentomics have emerged as powerful pillars of LB. DNA methylation, often analyzed via bisulfite conversion or increasingly popular bisulfite-free and immunoprecipitation-based techniques (e.g., MeDIP-Seq), provides stable biomarkers for longitudinal monitoring and tissue-of-origin identification [104,105,106]. Concurrently, cfDNA fragmentomics offers a non-invasive window into tumor biology by exploiting the characteristic shortening of tumor-derived DNA fragments (<160 bp) and altered fragmentation patterns to enhance diagnostic accuracy [107,108,109,110].

ctDNA analysis has become a cornerstone of personalized BC management, enabling real-time monitoring of therapeutic response and the identification of actionable mutations, such as PIK3CA and ESR1, to guide targeted interventions [111,112,113]. Its clinical utility is particularly profound in the detection of MRD, often identifying recurrence months before clinical or radiological evidence appears [114,115]. Supported by ASCO guidelines and FDA-approved companion diagnostics, ctDNA is transforming clinical practice by facilitating early adaptation of treatment strategies, overcoming drug resistance, and ultimately improving patient outcomes. Despite these challenges, ctDNA remains one of the most promising LB analytes for early-stage BC [116]. Tumor-specific mutations, methylation signatures, and fragmentation patterns may be detectable before clinical relapse becomes evident, providing opportunities for earlier intervention and more accurate risk stratification [22]. However, the low ctDNA fraction typically observed in early-stage disease continues to represent a major obstacle to widespread clinical implementation.

The clinical utility of ctDNA assays is highly dependent on the specific clinical context in which they are applied. Currently, the strongest evidence supports their use for molecular profiling, identification of actionable genomic alterations, detection of MRD, longitudinal monitoring of treatment response, and early identification of resistance-associated mutations that enable timely therapeutic adaptation [117]. In these settings, ctDNA provides clinically actionable information that complements conventional diagnostic approaches and has already been incorporated into selected areas of oncology practice [118,119]. Recent clinical evidence further illustrates this transition from a promising biomarker to a clinically actionable tool. Notably, the SERENA-6 trial demonstrated that longitudinal ctDNA monitoring could identify emerging ESR1 mutations before radiological progression in patients with metastatic HR-positive BC, enabling ctDNA-guided treatment switching to camizestrant and significantly delaying disease progression [118].

Beyond treatment guidance, ctDNA-based technologies are also being explored for population-level early cancer detection through Multi-Cancer Early Detection (MCED) assays [120]. The recently reported NHS-Galleri trial (NCT05611632) represents a major milestone in this field [121]. Although the study did not fully meet its primary endpoint, it demonstrated evidence of a favorable stage shift, with fewer stage IV cancers and a greater proportion of cancers detected at stages I and II, highlighting the potential of MCED technologies to improve earlier cancer diagnosis while emphasizing the need for further optimization and validation [120,121].

Despite these encouraging developments, ctDNA should not currently be considered a stand-alone screening modality or a replacement for tissue biopsy and conventional imaging [122]. In early-stage BC, tumors often release only minute quantities of ctDNA into the circulation, limiting analytical sensitivity despite continuous technological advances. Moreover, biological variability, the lack of standardized pre-analytical and analytical workflows, and unresolved cost-effectiveness issues continue to restrict routine clinical implementation [121]. Consequently, current evidence supports the selective use of ctDNA assays when the results are expected to directly influence patient management, whereas broader implementation for population-based screening and early cancer detection should await further prospective multicenter studies demonstrating both clinical utility and cost-effectiveness [117,121].

The limitations underlying these restricted clinical applications are largely attributable to the technical and biological challenges associated with detecting extremely low levels of ctDNA in early-stage disease. The transition of ctDNA monitoring to early-stage BC settings presents significant diagnostic challenges, primarily due to the minimal levels of tumor-derived DNA often circulating in the bloodstream [115,123]. Given these low concentrations, analytical platforms frequently encounter difficulty in distinguishing true pathogenic signals from background genetic noise, which inherently elevates the risk of false-positive results. Refinement of these assays is therefore essential to improve both sensitivity and specificity, ensuring that longitudinal surveillance provides clinically reliable data while minimizing the ambiguity associated with detecting minute genomic alterations.

## 5. Extracellular Vesicles

In addition to CTCs and CtDNA, EVs have emerged as a key component of LBs. EVs are a heterogeneous population of cell-derived structures originating from the membrane [124]. They are broadly classified into exosomes and microvesicles (MVs) based on their biogenesis pathways. Exosomes are generated through the endosomal pathway, whereas MVs are shed directly from the plasma membrane [125,126,127]. However, MISEV2018 guidelines highlight that assigning EVs to specific biogenesis pathways remains challenging, and recommend operational classifications based on physical characteristics, biochemical composition, or cellular origin [128]. Importantly, EV biogenesis shapes cargo composition through selective sorting within trafficking pathways that define the molecular content of intraluminal vesicles [129]. This biologically active cargo, such as proteins, miRNA and other non-coding RNAs, is horizontally transferred by the EVs, making them important mediators of intracellular communication [127,130,131]. More specifically, cancer cells have been found to release higher numbers of EVs than normal cells and their molecular cargo may contribute to tumor progression, metastasis, and therapy resistance [127]. Beyond their role as biomarkers, EVs actively participate in tumor progression by modulating immune responses and preparing pre-metastatic niches, highlighting their importance as functional drivers of disease evolution.

Certain EV associated proteins have been widely investigated as potential biomarkers in BC, with evidence supporting roles in early detection, disease progression, and therapy response. Among them, proteins such as Del-1, MUC1 and CEA have been reported to distinguish BC patients from healthy individuals, while Del-1 appears to be particularly relevant in early-stage disease [132,133,134]. Specifically, Del-1 has been found to be higher in early-stage BC [135].

In the context of disease progression and metastasis, exosomal EDIL3, has been shown to enhance BC progression and invasion via integrin-FAK signaling, suggesting a role in metastatic processes [136]. Moreover, phosphoproteomic profiling of plasma-derived EVs revealed distinct phosphorylation patterns associated with tumor progression and metastasis, with SRC tyrosine kinase signaling identified as a central regulatory pathway [137]. Consistent with these findings, EV surface protein panels including CA15-3, CA125, CEA, HER2, EGFR, PSMA, EpCAM, and VEGF have been shown to distinguish metastatic from non-metastatic BC and healthy controls, with PSMA emerging as an important factor associated with disease progression [138]. Differential enrichment of VEGF, EpCAM and EGFR, HER2/ERBB2, EPHA2, and GRB7 further reflects subtype-specific biological pathways and tumor heterogeneity [139]. EV cargo may also reflect mechanisms underlying therapeutic response and resistance. BC derived MVs carrying Hsp90-bound VEGF have been found to promote angiogenesis and reduce sensitivity to Bevacizumab [140]. Collectively, these findings highlight the multifaceted role of EV-associated proteins as non-invasive biomarkers for early detection, disease monitoring, metastatic progression, and therapeutic response in BC.

Beyond proteins, EV-associated miRNAs have also been implicated in BC pathogenesis and progression. In general, EV RNA is highly disease specific and may be used for early cancer detection and even tumor type classification [135]. A recent study demonstrated that HER2+ and CD24+ plasma EV subpopulations contain miRNA biomarkers capable of distinguishing benign breast lesions from early-stage BC, including DCIS and stage I disease [141]. Importantly, these findings highlight the value of analyzing specific EV subpopulations rather than total circulating EVs. Given EV heterogeneity, distinct vesicle subsets may capture different aspects of tumor biology. Immunomagnetic isolation of HER2+ and CD24+ plasma EVs revealed distinct and complementary miRNA profiles, with limited overlap between the two fractions. Combining biomarkers from both EV populations enabled accurate classification of malignant versus benign BI-RADS 4 breast lesions. Thus, EV sub-fractionation based on surface markers may improve the clinical value of EV biomarkers by enabling more targeted analysis of tumor vesicle populations [141].

Consistent with these findings, numerous EV-associated miRNAs have been linked to BC development and early diagnosis, including miR-1246, miR-375, miR-222, miR-155, miR-19a, miR-181b, miR-373, miR-223-3p, miR-24 and miR-21. Among these, miR-21 specifically has been correlated with tumor size and circulating tumor cells [132,134]. Moreover, miR-21 as well as miR-10b and miR-105 have been associated with increased invasion and endothelial disruption, while elevated levels of miR-105 in particular have also shown potential for predicting metastasis and patient outcome [127]. Other miRNAs that are associated with metastatic progression and poor prognosis include: miR-106b-5p, miR-18a-5p, miR421, miR128-1 and miR128-2 [142,143].

Among non-coding RNAs, elevated exosomal lncRNAs H19, HOTAIR and SNHG14 have been associated with BC as well. Specifically, HOTAIR is linked to poor prognosis and chemotherapy response, while SNHG14 is associated with trastuzumab resistance in HER2+ BC [134]. Similarly, several exosomal mRNAs such as GRM1, S100A8, GRIK1, CSTA, H6PD, IGF2BP1, TPT1 and MDM4 can distinguish BC patients from healthy controls with ~92% diagnostic accuracy. Elevated GSTP1 mRNA has also been associated with drug resistance, while TK1 and CDK9 mRNAs are associated with poor response to palbociclib in HER2+ BC [132,134].

Another promising class of biomarkers in BC LB is lipids. The lipid composition of EVs reflects the molecular features of the tumor cells of origin and includes bioactive lipids involved in tumor progression and metastasis. More specifically, BC derived EVs have been shown to contain elevated levels of sphingolipids, while metastatic BC EVs were especially enriched in phosphatidylserine (PS) and hexosylceramides (HexCer) [144]. Together, these findings highlight the potential of EV lipidomics as a complementary biomarker approach in BC detection and monitoring [144].

Furthermore, EV-derived DNA remained detectable and suitable for PCR and mutation analysis even under unfavorable storage conditions, likely due to the protective effect of the lipid bilayer. In addition, mutations identified in EV DNA closely matched those detected in tumor tissue, supporting the use of EVs as reliable carriers of tumor-derived genetic information [145]. Conflicting findings have also been reported, as some suggest that isolating EVs for DNA-based biomarkers does not improve cancer detection, as both ctDNA and mtDNA are more effectively and sensitively quantified in total patient plasma than in EV-enriched fractions [133]. However, another study found that EV-DNA and ctDNA provide complementary but distinct information due to their different biological origins. While ctDNA predominantly originates from apoptotic and necrotic tumor cells, EV-DNA is associated with vesicles actively secreted by viable cells. As a result, they appear to capture different aspects of tumor evolution [146]. EV-DNA was found to potentially better capture therapy resistance related alterations, such as ESR1 mutations that are associated with shorter survival, and ctDNA reflects early truncal mutations, such as PIK3CA which show prognostic value [147]. Overall, EV-DNA appears to be a promising complementary biomarker to ctDNA for monitoring treatment response and endocrine resistance in metastatic BC [147]. Representative EV-associated biomarkers investigated in BC LB, together with their principal clinical associations, are summarized in Table 1.

Beyond their value as diagnostic and prognostic biomarkers, EVs act as key mediators of intercellular communication and play a central role in cancer progression and metastasis. They promote tumour proliferation, invasion, and EMT, thereby facilitating metastatic spread to distant organs. In addition, tumor derived EVs contribute to immune evasion and pre-metastatic niche formation by modifying the TME to support metastatic colonization [150].

In particular, TNBC EVs have been found to promote tumor growth through activation of signalling pathways such as PI3K/AKT and by reprogramming stromal cells, including cancer-associated fibroblasts and mesenchymal stem cells (MSCs) [151]. By interacting with stromal cells, including cancer-associated fibroblasts and mesenchymal stem cells, EVs stimulate fibroblast activation, angiogenesis, and stromal remodelling, while also enhancing cancer stemness and chemoresistance [151,152,153]. Moreover, they contribute to the establishment of pre-metastatic niches by modifying distant organ microenvironments and enhancing vascular permeability, thereby facilitating metastatic colonization of organs such as the lungs, brain, and bone [151,154,155]. Interestingly, some research suggests that certain chemotherapies may unintentionally promote metastasis by causing tumor cells to release EVs that create a pro-inflammatory, metastasis-friendly environment in the lungs [156]. Furthermore, although TNBC derived EVs have been shown to exert the strongest immunomodulatory effects, EV mediated immune remodeling has also been observed in other BC subtypes such as EB+, suppressing anti-tumor immune responses and promoting the accumulation of immunosuppressive cell populations within the TME. Through the transfer of molecules such as PD-L1 and regulatory miRNAs, EVs reduce T-cell activity, promote M2 macrophage polarization, and support stromal remodelling, thereby facilitating tumor progression and metastasis [151,155,157,158,159,160].

However, the immunomodulatory effects of BC derived EVs are complex and EVs are not uniformly immune suppressive, as some EV populations have been associated with immune activation. TNBC EVs have been shown to promote pro-inflammatory macrophage phenotypes, enhance T-cell infiltration, and correlate with improved patient survival, highlighting their potential to support anti-tumor immune responses under specific conditions [161]. Conversely, EVs released under hypoxic conditions promote EMT, invasion, expansion of progenitor cell populations, and systemic immunosuppression, while EV-associated chemokines contribute to immune cell recruitment and pre-metastatic niche formation in distant organs [162]. More findings suggest that EVs are able to modulate anti-tumor immune responses in a subtype-dependent manner and contribute to tumor progression through immune remodelling within the TME [163]. Collectively, these findings highlight the multifaceted role of EVs in BC, functioning not only as active mediators of immune modulation, stromal remodeling, and pre-metastatic niche formation, but also as promising biomarkers for both early diagnosis and monitoring of BC due to their accessibility in biological fluids, stability, and rich molecular cargo that reflects tumor characteristics, making them valuable for minimally invasive sampling. heir biomarker potential is further supported by the structural stability provided by the lipid bilayer, which protects their molecular cargo from degradation in circulation and enables stable detection in plasma [137,148,164].

Nevertheless, EVs’ clinical applications face several challenges, including low vesicle yield, complex isolation procedures, difficult and non-standardized purification methods, which often result in variable recovery rates and contamination with non-vesicular components [134,164,165]. Another important limitation is that early-stage tumors may produce only minimal EV signals, which reduces detection sensitivity [166]. Furthermore, tumor-derived EVs represent only a small fraction of the total circulating EV population, making them difficult to identify among abundant EVs released in circulation by normal cells [34]. To overcome these challenges, recent ISEV guidelines highlight the importance of standardized reporting frameworks and comprehensive EV characterization strategies to improve reproducibility across studies. Since tumor-derived EVs constitute only a small proportion of circulating EVs, complementary separation techniques and single-EV analytical approaches are often required, as no single isolation method can simultaneously provide high recovery and high specificity [134,164].

Although advanced methodologies, such as asymmetric flow field-flow fractionation, density gradient ultracentrifugation, and single-EV flow cytometry, offer improved EV specificity and characterization, their implementation in routine clinical laboratories is still limited due to technical complexity, need for specialized instrumentation, and restricted scalability. Therefore, successful clinical translation will depend on achieving an appropriate balance between high-purity EV isolation and practical, scalable workflows suitable for routine diagnostic applications [34,165]. Overall, continued development of accessible and reproducible EV isolation and analysis platforms, together with improved biological understanding and clinical validation, will be essential for realizing the potential of EVs as minimally invasive biomarkers in future precision medicine applications.

## 6. Circulating RNA, Proteins and Emerging Blood-Based Biomarkers

Beyond ctDNA, CTCs, and EVs, several additional blood-based biomarkers have shown potential as LB tools in BC, providing minimally invasive disease monitoring and potentially enhancing diagnostic and prognostic accuracy. Numerous miRNAs previously identified in EV-associated cargo, including miR-10b, miR-21, miR-34a, miR-155, and miR-210 have also been detected as circulating cell-free biomarkers in serum. Their elevated levels were associated with BC progression, EMT and metastasis, supporting the broader clinical relevance of miRNA-based LB approaches [167,168]. Among them, miR-155 has been investigated quite extensively as a very promising blood biomarker. Studies have reported significantly elevated miR-155 levels in patients with early-stage BC compared with healthy controls, supporting its potential utility for early detection [169]. In addition, a recent meta-analysis reported a sensitivity of 93% and specificity of 85% [170]. Moreover elevated miR-155 levels have also been associated with lymph node metastasis, recurrence, and therapy resistance [170]. Another frequently studied circulating miRNA is miR-21, which is consistently upregulated in BC patients. It has been associated with tumor progression, invasion, metastasis, and reduced survival, while a recent meta-analysis reported a sensitivity of 91% and specificity of 85% for BC detection. However, its diagnostic accuracy may be further enhanced when miR-21 is combined with other miRNAs [171].

Additional circulating miRNAs have demonstrated diagnostic, prognostic, and predictive value. For example, miR-127-3p, miR-148b, miR-409-3p, miR-652, and miR-801 have been associated with early-stage BC, whereas miR-202 and miR-10b have been linked to poor clinical outcomes. Other miRNAs, including miR-18b, miR-103, miR-107, and miR-652, have shown potential for relapse prediction in TNBC, while miR-125b, miR-122, and miR-375 have been associated with chemotherapy response and disease recurrence [172]. More recent studies have further linked circulating miRNAs to specific biological and clinical features of BC. For instance, miR-1246 has been associated with brain metastasis, whereas reduced levels of miR-206 and miR-199a-3p have been linked to distant metastatic spread. Distinct miRNA profiles have also shown potential for molecular subtype classification and personalized treatment strategies [173]. Although individual miRNAs such as miR-155 and miR-21 have shown promising diagnostic performance, accumulating evidence suggests that multi-miRNA panels may provide greater accuracy for BC detection. Several studies have reported sensitivities exceeding 90% using combinations of circulating miRNAs. However, no circulating miRNA panel has yet been clinically validated [174].

In addition to miRNAs, circulating lncRNAs are emerging as promising LB biomarkers. Among these, MALAT1 has been associated with tumor progression, lymph node metastasis, and poor prognosis, while AFAP1-AS1, PVT1, and RP11-218M22.1 have exhibited subtype-specific expression patterns [173]. Compared with miRNAs, circulating lncRNAs may provide more disease-specific information and have shown associations with distinct clinicopathological features. However, they are still at an earlier stage of clinical validation, with limited standardization across detection methods and a need for further large-scale studies to confirm their clinical utility [175].

In addition to the EV associated proteins discussed in the previous section, freely circulating serum and plasma proteins have also been investigated as LB markers in BC. Unlike EV-encapsulated proteins, these soluble proteins are not protected by a phospholipid bilayer and therefore exhibit different stability profiles and pre-analytical characteristics. Nonetheless, traditional circulating protein markers such as CEA, CA15-3, CA27-29, CA-125, HER2 extracellular domain (sHER2), and TPS can be detected in BC patients, but their usefulness for early-stage detection is limited by low sensitivity and specificity [175]. However they remain useful for prognosis and treatment monitoring [176]. Additionally, a lot of the protein markers reflect systemic immune responses and tumor immune interactions. Circulating IL-6, a pro-inflammatory cytokine involved in the acute-phase response, has been consistently associated with advanced disease stage [176]. Similarly, elevated levels of IL-8 and lipocalin-2 (LCN2) have been associated with tumor progression and poor clinical outcomes, whereas higher interferon-γ (IFN-γ) levels correlate with improved prognosis.

Additionally, soluble PD-L1 may reflect tumor immune evasion and response to immunotherapy [176]. Proteomic analyses of serum from HER2+ patients receiving neoadjuvant chemotherapy (NAC) identified distinct protein profiles enriched in patients with poor treatment response. In contrast, other circulating proteins, such as gelsolin (GSN) and immunoglobulin kappa constant (IGKC), have been associated with improved prognosis and chemotherapy sensitivity [177].

One of the most studied and well-established circulating protein markers is CA15-3. Elevated plasma CA15-3 levels have been associated with unfavourable clinical outcome, including shorter disease-free and metastasis-free survival, supporting its value as a circulating prognostic biomarker in BC [178]. Still, it shows limited utility for early detection and lack sufficient specificity, with variable performance across studies and BC subtypes [176]. In contrast, CCN1 was found to be significantly elevated in BC patients and demonstrated high diagnostic accuracy, outperforming conventional serum markers such as CA15-3 and CEA, suggesting its potential utility in future LB panels for early diagnosis [179]. However, large proteomic studies have yielded conflicting results, highlighting the need for further validation of circulating protein biomarkers [180]. It is important to note that the utility of such circulating proteins for early detection remains limited due to insufficient sensitivity and specificity when used individually [174].

In addition to circulating nucleic acids and proteins, tumor educated platelets (TEPs) have emerged as a noteworthy LB component. Unlike tumor-derived biomarkers, TEPs represent alterations that arise through interactions with cancer cells and the TME. These interactions have been found to modify platelet RNA profiles, which can distinguish patients from healthy individuals and provide information regarding tumor origin and molecular characteristics [181]. Importantly, a TEP-based classifier achieved 91% accuracy in patients with stage I/II BC, supporting the potential of platelet RNA profiling as a blood-based screening approach for early disease detection [182]. Notably, the diagnostic performance of RNAs derived from TEPs appears to be independent of tumor stage, molecular subtype, BRCA mutation status, and breast density [183]. TEP RNA profiles were able to identify specific BC characteristics, including HER2-amplified and TNBC tumors [184]. Furthermore, platelets contain angiogenesis-related proteins, such as VEGF, ANGPT-1, MMP2, PF-4 and PDGF, whose altered levels may reflect tumor activity [181].

These findings highlight the potential of platelet transcriptomic and proteomic profiles as non-invasive biomarkers in BC, particularly when integrated with machine-learning approaches for improved cancer detection, classification, and treatment monitoring [184]. Nevertheless, the clinical applicability of TEPs remains uncertain. Subsequent validation studies have shown that platelet mRNA profiles are highly sensitive to pre-analytical and technical variables, including sample processing procedures, highlighting the need for further methodological optimization before TEP RNA profiles may be reliably implemented for BC detection [185].

Another important class of blood-based biomarkers is autoantibodies (AAbs), which arise from immune responses against tumor-associated antigens and may be detectable before clinical diagnosis. They represent promising early detection biomarkers in BC due to their stability, prolonged circulation, and ability to reflect early tumorigenic changes, with multi-autoantibody panels offering improved diagnostic performance over single markers [186]. Several studies have identified AAbs against proteins encoded by oncogenes and tumor suppressor genes, including anti–human endogenous retrovirus-K (HML-2), which distinguish patients with invasive disease and ductal carcinoma in situ from healthy individuals [187]. Diagnostic performance may be further improved by combining AAbs with established markers, such as CA15-3, or by constructing multi-autoantibody panels that show higher sensitivity and specificity than single antibodies [187].

Metabolomic profiling in blood samples has shown promise for early BC detection, with studies identifying metabolic signatures, such as caprylic acid and hypoxanthine, that distinguish patients with early-stage disease from healthy controls, highlighting their potential as non-invasive diagnostic biomarkers [188]. Collectively, alternative samples provide complementary information to blood-based assays and contribute to a more comprehensive assessment of BC biology [189]. Despite the promising results, LBs based on non-blood samples remain clinically limited by variability in sample collection, processing, storage, and analysis, as well as technical challenges, high costs, and lack of standardization, with false positives remaining a concern. Further validation and standardized protocols are needed before they can be routinely used in clinical practice [189]. The main strengths and limitations of emerging blood-based biomarkers in early BC are summarized in Table 2.

Overall, while circulating RNA and protein-based biomarkers are promising components of LB in BC, offering minimally invasive insight into tumor biology and disease progression their clinical utility is limited by suboptimal specificity and influence from physiological and inflammatory processes, which reduces their reliability when used individually. Therefore, their greatest potential likely lies in multi-marker and multi-omics approaches, particularly biomarker panels that integrate different classes of circulating components, to improve sensitivity, specificity, and clinical applicability in early BC detection and monitoring.

## 7. Clinical Applications of Liquid Biopsy in Early Breast Cancer

Despite substantial advances in analytical sensitivity, the translation of LB into routine management of early BC remains challenging [190]. Unlike advanced disease, early-stage tumors release only limited amounts of tumor-derived material into the circulation, resulting in weak and often intermittent biological signals [191,192]. Consequently, current clinical efforts are primarily focused on three areas in which LB may provide meaningful benefit: population screening, detection of minimal residual disease (MRD), and prognostic risk assessment [190].

At present, the clinical application of LB in early-stage BC remains experimental and is not integrated into routine standard-of-care management guidelines [190]. Current clinical research investigates its utility across three distinct domains: asymptomatic screening, MRD detection, and risk stratification. For asymptomatic screening, next-generation multi-cancer early detection (MCED) assays utilize tumor-specific methylation profiles or cfDNA fragmentation signatures to identify breast malignancies prior to the radiological visualization of structural lesions [193]. A prominent example of this framework is the Circulating Cell-free Genome Atlas (CCGA) study, which evaluated a targeted methylation-based assay for multi-cancer screening [193].

However, the biological scarcity of circulating biomarkers in localized, early-stage disease presents a severe technical challenge. Due to the profoundly low cellular shedding rates of small, non-metastatic breast lesions into the bloodstream, the clinical sensitivity of these assays remains highly restricted in early settings [35]. For instance, in the CCGA cohort, while the assay demonstrated a near-perfect specificity of 99.5%, its sensitivity for stage I BC was significantly lower than for advanced stages, highlighting a pronounced risk of false-negative results that currently limits its utility as a standalone screening tool [193].

Recent technological developments have expanded screening strategies beyond mutation-based analyses toward broader characterization of circulating nucleic acids. In particular, methylation profiling, fragmentomic signatures, and machine learning–driven multi-cancer early detection (MCED) platforms have emerged as promising approaches for identifying tumor-associated patterns in plasma [194]. These methodologies aim to overcome the limited abundance of ctDNA in localized disease by exploiting genome-wide biological features rather than relying solely on individual genetic alterations [195]. Nevertheless, despite encouraging specificity, their performance in stage I BC remains suboptimal, emphasizing the persistent challenge of detecting tumors with minimal systemic dissemination [194].

Beyond initial diagnosis, research is heavily focused on the detection of MRD following definitive primary interventions, such as surgical resection or neoadjuvant chemotherapy [196]. Longitudinal tracking of patient-specific cfDNA mutations in plasma allows for the identification of molecular recurrence before macro-metastatic lesions manifest radiologically via conventional imaging protocols [197]. For instance, personalized ctDNA monitoring has been shown to detect molecular relapse a median of 8.9 months ahead of routine surveillance scans [196]. Although this high-sensitivity tracking identifies patients at an elevated risk of recurrence when the systemic tumor burden is at its biological minimum, its prospective clinical utility remains unverified. It is currently unproven whether initiating secondary systemic therapies during this molecular-only recurrence phase translates into a definitive overall survival benefit. Consequently, therapeutic adjustments based strictly on post-operative MRD status remain investigational and are restricted to clinical trial settings [190].

Increasing evidence suggests that single-analyte approaches may not adequately capture the biological complexity of early BC. Consequently, recent investigations have shifted toward multimodal LB strategies that integrate ctDNA with CTCs, EVs, circulating RNAs, protein markers, and host-derived immune signals [198,199]. By combining complementary sources of information, these platforms may enhance analytical sensitivity while maintaining specificity, particularly in tumors characterized by low shedding rates [198]. Although such integrated approaches remain largely investigational, they are increasingly viewed as a potential avenue for overcoming the limitations associated with individual biomarkers [200].

The quantification of circulating biomarkers provides established prognostic data for baseline and post-treatment risk stratification in early-stage settings [35]. Prospective evidence confirms that patients presenting with persistently detectable ctDNA post-surgery or elevated CTC counts exhibit significantly shorter disease-free survival (DFS) and a heightened risk of distant recurrence [201]. For example, data from the BRE12-158 trial demonstrated that the presence of ctDNA after neoadjuvant chemotherapy in early-stage TNBC is strongly prognostic of distant DFS failure. Circulating biomarkers effectively segregate high-risk from low-risk populations [199].

The prognostic significance of circulating biomarkers may also differ across BC subtypes. In TNBC, post-treatment ctDNA positivity has consistently been associated with an increased likelihood of distant relapse and inferior survival outcomes [202]. Similarly, residual circulating biomarkers following neoadjuvant therapy appear to identify patients at particularly high risk despite achieving favorable conventional clinicopathological responses [203]. In hormone receptor-positive (HR+) tumors, ctDNA monitoring may provide insight into prolonged periods of minimal residual disease and delayed recurrence patterns characteristic of this subtype. However, there is currently no consensus data proving that utilizing these molecular trajectories to guide treatment escalation, such as the empirical addition of adjuvant systemic therapies, or treatment de-escalation may safely improve patient clinical outcomes outside of clinical trials [190,201]. Representative clinical studies evaluating the current utility and limitations of LB in early BC are summarized in Table 3.

Among currently explored applications, MRD detection appears to represent the most clinically mature use of LB in early BC, owing to its ability to identify molecular recurrence months before conventional imaging [114,197]. In contrast, population-wide screening remains constrained by the extremely low abundance of circulating biomarkers in localized disease, while prognostic applications continue to face challenges regarding clinical actionability [204]. Therefore, although LB has demonstrated substantial prognostic and monitoring capabilities, its integration into therapeutic decision-making remains an area of active investigation. The major clinical applications of LB in early BC, together with their principal benefits, limitations, and current level of clinical maturity, are summarized in Table 3.

Collectively, current evidence supports LB as a valuable adjunct to, rather than a replacement for, conventional imaging and tissue-based diagnostics in early BC. While circulating biomarkers have demonstrated considerable promise for screening, MRD detection, and prognostic stratification, significant biological and technical challenges continue to limit their routine clinical implementation. Consistent with current international consensus recommendations, tissue-based histopathology and conventional imaging remain the cornerstone of diagnostic, staging, and therapeutic decision-making in early BC [190]. The transition of LB from a prognostic and monitoring tool to a clinically actionable platform will depend on evidence generated from ongoing large-scale prospective interventional studies [39].

Future progress will likely rely on the integration of multiple biomarker classes, advanced computational models, and multimodal analytical frameworks capable of improving sensitivity while preserving clinical specificity. Most importantly, prospective trials must demonstrate that biomarker-guided therapeutic interventions lead to measurable improvements in survival outcomes and treatment optimization compared with current standard management approaches. Until such evidence becomes available, LB should be regarded as a highly promising but still evolving component of precision oncology in early BC.

## 8. Technological Challenges and Future Perspectives in Liquid Biopsy

The clinical implementation of LB in early-stage BC faces a major, unresolved challenge regarding the trade-off between assay sensitivity and clinical specificity [190,205]. In early-stage settings, the absolute quantity of tumor-derived DNA in the bloodstream is extremely scarce, frequently constituting less than 0.01% of the total cfDNA pool [35]. Attempts to optimize assay sensitivity to capture these minute traces inevitably amplify technical and biological background noise, which drastically compromises specificity. While Clonal Hematopoiesis of Indeterminate Potential (CHIP) represents a well-documented biological driver of false-positive results, the spectrum of confounding factors extends far beyond hematopoietic mutations [190]. Infrequently discussed yet critical vulnerabilities include sequencing artifacts and PCR errors, where nucleotide substitutions induced during PCR amplification or high-throughput NGS processes may mimic low-frequency tumor mutations. To mitigate this biological interference, contemporary diagnostic pipelines routinely incorporate the sequencing of matched peripheral blood mononuclear cells (PBMCs) or genomic DNA from patient-specific leukocytes, allowing bioinformatics filters to effectively identify and subtract background hematological variants from true tumor-derived alterations [190]. Additionally, sample contamination during high-sensitivity runs frequently generates false calls, while confounding background cfDNA signals from non-malignant systemic processes such as tissue inflammation, physical trauma, or benign cellular turnover release substantial amounts of wild-type genomic DNA into the circulation, effectively masking rare BC signals [205].

Furthermore, pre-analytical variables remain a substantial barrier to cross-platform reproducibility and widespread clinical translation [39]. The prevention of leukocyte lysis requires immediate, specialized processing of standard EDTA blood collection tubes. Failure to execute rapid centrifugation prompts the release of high-molecular-weight genomic DNA from healthy white blood cells, which dilutes the rare BC DNA fraction and causes false-negative outcomes [192]. Alternative cell-stabilizing preservative tubes mitigate this issue but frequently introduce distinct chemical fixation artifacts that interfere with downstream enzymatic assays. Consequently, the field suffers from profound inter-laboratory variability. The lack of international reference standards, coupled with the utilization of diverse NGS platforms and proprietary, non-standardized bioinformatics pipelines across different centers, creates a major bottleneck that prevents LB from meeting regulatory benchmarks for routine clinical diagnostic adoption [39,190]. Harmonization of sample collection protocols, sequencing workflows, quality-control metrics, and bioinformatic pipelines will be essential before LB achieves regulatory approval and widespread clinical adoption [14]. To further evaluate these parameters, the methodological profiles, analytical sensitivities, and distinct technical limitations of prominent commercial ctDNA assays are summarized in Table 4.

Despite significant analytical advances, economic and logistical barriers continue to hinder widespread implementation [15]. Ultra-deep sequencing, multi-omics profiling, and AI-driven analytical frameworks require substantial infrastructure, specialized personnel, and high computational resources [23,206]. Consequently, translating these technologies from highly specialized research centers to routine clinical practice remains a major challenge, particularly in resource-limited healthcare systems [15]. The major technological barriers currently limiting the clinical implementation of LB, together with emerging strategies designed to overcome these limitations, are summarized in Table 5.

To address the analytical limitations detailed above, current frameworks are shifting from single-mutation tracking toward comprehensive multi-omics integration [192]. Analyzing independent genetic alterations in isolation often fails to reliably differentiate true oncology signals from background artifacts or CHIP variants. To resolve this specificity dilemma, next-generation platforms simultaneously evaluate genetic mutations alongside cfDNA epigenetic methylation profiles, fragmentomics, and circulating protein signatures [193]. In early-stage breast oncology, mapping tumor-specific methylation patterns is highly valuable for suppressing biological background noise, as malignant cells harbor distinct tissue-specific epigenetic imprints [195]. Combining these multi-layered biological inputs with BC-specific protein markers may improve clinical sensitivity, mitigating the false-negative risks typical of low-shedding tumors [198].

Crucially, processing these multi-parametric datasets necessitates a departure from traditional statistical thresholds [192]. This computational shift requires the adoption of advanced Artificial Intelligence (AI) and machine learning architectures [193]. However, the current literature frequently overstates the capabilities of these computational models. Assertions that AI frameworks may effortlessly bypass normal tissue shedding lack robust clinical validation [192,195]. In reality, machine learning models face major methodological limitations that impede clinical translation, with data scarcity and severe overfitting representing the primary [198]. Training deep neural networks requires massive, highly curated datasets which are notoriously limited in early-stage BC cohorts [193]. Consequently, algorithms that perform exceptionally well on internal training data frequently fail when exposed to heterogeneous real-world populations [198]. Nevertheless, when trained and externally validated using large multicenter datasets, machine learning models may facilitate the integration of complex multi-analyte information that would be difficult to interpret through conventional statistical approaches [15].

This diagnostic vulnerability is further compounded by a profound lack of mechanistic explainability, a limitation widely described as the black box problem [193]. The inherent opacity prevents clinicians from understanding the underlying algorithmic calculations, which fundamentally hinders clinical trust [190,192]. Finally, cohort-to-cohort inconsistency remains highly prevalent because models optimized for a specific population rarely demonstrate reproducible accuracy externally [198]. These clinical failures stem from minor variations in sample preparation protocols combined with technical discrepancies between different sequencing platforms and bioinformatics pipelines [39,190]. 

The optimization of future perspectives in LB depends on its capacity to address intra-tumoral heterogeneity in early-stage BC. This biological phenomenon frequently reduces standard blood draws to an incomplete systemic average [192]. To transition from a purely descriptive modality to a clinically actionable instrument, LB frameworks must be structurally linked to concrete clinical goals [39].These target domains include objective patient stratification, early detection, MRD monitoring, and precise treatment selection [190]. This concept may be particularly relevant in early-stage BC, where low-abundance biomarkers often fail to fully represent intratumoral heterogeneity [207].

The primary innovation in this domain relies on an integrated model termed spatial-single-cell-informed biomarker discovery, which synthesizes three complementary axes of biological information [192]. Within this paradigm, cfDNA assays provide systemic longitudinal dynamics to monitor disease evolution over extended timelines [35]. Concurrently, regional tissue profiles supply the necessary spatial topography to map the precise physical architecture of the primary tumor ecosystem. Finally, the isolation and sequencing of rare CTCs achieve single-cell resolution, uncovering low-frequency cellular subpopulations that are typically lost in bulk plasma sequencing frameworks [35,192].

This multi-layered approach facilitates the longitudinal tracking of specific subpopulation shifts rather than treating malignancy as a static entity [35]. For instance, such multi-dimensional modeling may enable future adaptive therapeutic strategies by detecting the sub-clinical emergence of a HER2^+^ or drug-resistant sub-clone within a patient whose primary lesion was characterized as predominantly hormone-receptor-positive [197]. By mapping these shifting cellular states onto an integrative ecosystem profile, clinicians may eventually possess the capacity to identify emerging resistance mechanisms in real time [35]. This optimized workflow may permit personalized therapeutic adjustments months before a macro-metastatic relapse becomes physically visible on routine surveillance scans [197]. Until large-scale prospective randomized trials validate these computational frameworks, however, such multi-omics applications remain strictly investigative [39,190].

## 9. Comparative Evaluation of Liquid Biopsy Biomarkers for Early Breast Cancer

Although substantial progress has been achieved in the development of LB technologies, no individual circulating biomarker currently provides the combination of sensitivity, specificity, reproducibility, and clinical robustness required for routine population-based screening of early BC [208]. Rather than competing approaches, the major liquid biopsy analytes should be regarded as complementary sources of biological information, each reflecting distinct aspects of tumor initiation, progression, and host–tumor interactions [15].

Among currently available biomarkers, ctDNA offers one of the highest levels of analytical specificity owing to its ability to identify tumor-specific genetic and epigenetic alterations, including somatic mutations, DNA methylation signatures, and fragmentation profiles [97]. Continuous advances in digital PCR, NGS, and fragmentomic analyses have substantially improved analytical sensitivity [209]. Nevertheless, the clinical performance of ctDNA remains intrinsically constrained by the biological characteristics of early-stage disease. Small tumor volume, limited vascularization, low apoptotic and necrotic activity, and restricted biomarker shedding frequently result in extremely low ctDNA fractions that approach the limits of analytical detection [15,210]. Consequently, although ctDNA has demonstrated considerable clinical value for molecular profiling, minimal residual disease detection, recurrence monitoring, and therapeutic guidance, its application as an isolated screening biomarker for asymptomatic early BC remains limited [211].

CTCs provide fundamentally different information by enabling direct characterization of intact malignant cells [212]. Unlike cell-free analytes, CTCs preserve cellular morphology, protein expression, genomic alterations, and functional characteristics, thereby offering unique opportunities to investigate epithelial–mesenchymal plasticity, metastatic competence, therapeutic targets, and mechanisms of treatment resistance [213]. However, these biological advantages are counterbalanced by major technical challenges. The extreme rarity of CTCs in early-stage disease, together with pronounced phenotypic heterogeneity and the dynamic loss of epithelial markers during EMT, substantially compromises isolation efficiency and contributes to false-negative detection [214]. Although CTC enumeration has established prognostic value in metastatic BC, its clinical application for early detection remains restricted by these biological and methodological limitations.

EVs represent an attractive alternative because they are actively secreted by viable tumor cells and carry a remarkably diverse molecular cargo, including DNA, RNA, proteins, lipids, and metabolites [215]. Their phospholipid bilayer protects these biomolecules from enzymatic degradation, conferring greater stability than many circulating analytes and facilitating comprehensive molecular characterization from a single biospecimen [216]. Furthermore, EVs are increasingly recognized as active mediators of tumor progression rather than passive biomarkers, contributing to immune modulation, stromal remodeling, angiogenesis, and pre-metastatic niche formation [215]. Despite these advantages, their translation into routine clinical practice remains hampered by the absence of standardized isolation and purification protocols, variability in analytical methodologies, and difficulties in distinguishing tumor-derived vesicles from the abundant background of vesicles released by normal tissues [217].

Circulating RNAs, proteins, tumor-educated platelets, metabolites, and additional blood-based analytes further expand the spectrum of detectable tumor-associated signals [97]. Several circulating miRNAs, lncRNAs, protein panels, and platelet-derived signatures have demonstrated encouraging diagnostic performance, particularly when evaluated as multimarker panels rather than individual biomarkers [208]. However, many of these analytes exhibit limited disease specificity, considerable biological variability, and susceptibility to confounding inflammatory or physiological conditions [14,23]. Moreover, differences in sample processing, analytical platforms, normalization strategies, and biomarker cut-off values continue to hinder direct comparison among published studies and impede clinical standardization. Τo facilitate comparison among the major liquid biopsy biomarker classes, their principal biological targets, diagnostic performance, major strengths, limitations, and current level of clinical readiness are summarized in Table 6.

Collectively, the available evidence indicates that the principal limitation of current LB strategies is not solely analytical sensitivity but also the biological complexity of early breast cancer itself [218,219]. During the earliest stages of tumor development, restricted vascular access, minimal tumor burden, limited biomarker shedding, and efficient physiological clearance generate only weak systemic signals, irrespective of the analytical platform employed [15]. Consequently, further technological improvements alone are unlikely to fully overcome the intrinsic biological constraints associated with early disease detection.

Taken together, these observations suggest that future advances will most likely arise from integrated multi-analyte strategies rather than continued optimization of individual biomarkers. The combination of complementary genomic, epigenomic, transcriptomic, proteomic, metabolomic, and cellular information, supported by artificial intelligence and multi-omics analytical frameworks, has the potential to substantially improve diagnostic accuracy while providing a more comprehensive representation of tumor heterogeneity than any single biomarker alone [15,208]. Such integrative approaches are therefore expected to represent the next stage in the clinical evolution of LB for early BC detection.

## 10. Discussion

LB has emerged as a promising strategy for the early detection of BC, enabling the non-invasive assessment of tumor-derived components released into various biological fluids [9,208]. Nevertheless, despite substantial technological advances and an expanding repertoire of circulating biomarkers, early-stage disease remains particularly challenging to identify [220]. The fundamental obstacle lies in the biology of early tumor development itself. Small primary tumors release only limited quantities of tumor-derived material into the circulation, resulting in a weak systemic signal that is often difficult to distinguish from the physiological background [221]. Consequently, the analytical sensitivity required for reliable detection is considerably higher in early-stage disease than in advanced cancer [25].

The various classes of LB biomarkers provide distinct and complementary biological insights, yet each is associated with inherent limitations that constrain its clinical applicability [14]. CTCs offer direct cellular evidence of malignancy and allow phenotypic and molecular characterization of tumor cells [3]. However, their extreme rarity in early-stage BC significantly restricts their diagnostic utility. Moreover, EMT may lead to the downregulation of commonly targeted epithelial markers, thereby reducing detection efficiency and increasing the likelihood of false-negative findings [222].

In contrast, ctDNA has become one of the most extensively investigated biomarkers due to its ability to reveal tumor-specific genetic and epigenetic alterations [223]. Detection of mutations, methylation patterns, and fragmentation signatures has demonstrated considerable potential for early diagnosis [96]. Nevertheless, ctDNA concentrations are frequently exceedingly low in early-stage disease, while age-related clonal hematopoiesis may generate false-positive findings that complicate interpretation [18]. EVs represent another attractive biomarker source because of their remarkable stability and their ability to transport biologically active cargo, including nucleic acids and proteins [224].

Nevertheless, the clinical translation of extracellular vesicles is still hindered by the absence of universally accepted protocols for their isolation, purification, and molecular characterization [225]. Similarly, circulating RNAs, proteins, metabolites, and tumor-educated platelets have shown promise, but their relatively limited specificity often reduces their effectiveness when evaluated as individual biomarkers [25,208]. Moreover, many RNA and protein-based biomarkers may be altered in non-malignant physiological or inflammatory conditions, further complicating their application in population-based screening settings [226]. Despite the remarkable expansion of LB technologies, many studies continue to evaluate circulating biomarkers in isolation. Such an approach may not fully capture the biological complexity of early-stage BC, where tumor-derived signals are sparse, heterogeneous, and strongly influenced by both tumor-intrinsic and host-related factors.

Circulating biomarkers are not merely passive indicators of disease burden. Their abundance, composition, and biological relevance are strongly influenced by tumor intrinsic characteristics as well as by interactions with the surrounding microenvironment. The generation of circulating tumor-derived signals is profoundly influenced by dynamic TME interactions, including angiogenesis, vascular remodeling, immune surveillance, and stromal reprogramming [221,227]. Consequently, the circulating biomarker landscape reflects the underlying biological activity of the disease, including dynamic interactions between tumor cells and the TME, rather than tumor size alone. This may partly account for the considerable variability in LB profiles observed among tumors of comparable size [221]. The biological heterogeneity of BC further complicates biomarker detection, as distinct tumor regions and cellular subpopulations may contribute unequally to the circulating biomarker pool. Consequently, tumors with similar clinical characteristics may generate substantially different LB profiles.

Furthermore, biological processes such as EMT, early dissemination, and tumor cell dormancy may further influence both the quantity and composition of circulating biomarkers, contributing to the weak and heterogeneous systemic signals characteristic of early-stage disease [228]. A major challenge across all LB platforms is the distinction between true tumor-derived signals and biological noise. Blood contains a vast array of nucleic acids, proteins, extracellular vesicles, and cellular components originating from normal physiological processes. As a result, highly sensitive analytical platforms may inadvertently detect non-tumor signals, increasing the risk of false-positive findings. This challenge is particularly pronounced in screening settings, where even small reductions in specificity may result in unnecessary diagnostic investigations, increased psychological burden, and the risk of overdiagnosis.

Beyond biological complexity, several technical barriers continue to hinder widespread clinical implementation. Variability introduced during the pre-analytical phase, including differences in sample collection, processing intervals, storage conditions, and extraction methodologies remains a significant source of analytical inconsistency. Furthermore, differences among analytical platforms, bioinformatic pipelines, and reporting criteria contribute to variability across studies and limit reproducibility. The lack of universally accepted standards remains one of the principal obstacles to the incorporation of LB into routine clinical practice.

Despite the rapidly expanding body of literature, the overall level of clinical evidence supporting LB for early BC remains limited. Although numerous circulating biomarkers have demonstrated encouraging diagnostic performance in exploratory studies, relatively few have progressed to large prospective multicenter validation or routine clinical implementation. Most currently available evidence derives from retrospective analyses, single-center cohorts, or studies involving relatively small patient populations, limiting the generalizability of published findings. Furthermore, considerable methodological heterogeneity, including differences in patient selection, pre-analytical handling, analytical platforms, and biomarker cut-off values complicates direct comparison across studies and continues to hinder robust clinical standardization. Consequently, while current findings strongly support continued investigation, additional large-scale prospective multicenter clinical trials remain essential before LB can be routinely implemented for early BC detection.

Taken together, current evidence indicates that no individual biomarker consistently achieves the sensitivity, specificity, and level of clinical validation required for reliable early BC detection and routine clinical implementation. Rather than focusing on a single universal biomarker, future LB strategies may benefit from integrating complementary analytes that capture distinct dimensions of tumor biology. Combining genomic, transcriptomic, proteomic, cellular, and immune-related information could provide a more comprehensive representation of early disease than any individual biomarker alone. Finally, an important unresolved challenge is determining whether circulating biomarkers accurately reflect the full biological diversity of BCs. Spatial heterogeneity, clonal evolution, and temporal changes in tumor composition may all influence the circulating biomarker landscape. As tumors evolve, the relative contribution of individual cellular subpopulations to circulating biomarkers may also change, potentially affecting biomarker performance over time. Consequently, longitudinal monitoring and repeated sampling may prove more informative than single time-point measurements, particularly for tracking disease evolution, identifying emerging resistant clones, and refining patient risk stratification.

Despite encouraging progress, current clinical evidence remains insufficient to support the routine implementation of LB for population-based early BC detection. Most promising biomarkers still require validation in large prospective multicenter studies using standardized analytical workflows and harmonized reporting criteria. The successful translation of LB into routine clinical practice will require rigorous validation in large prospective cohorts, the establishment of standardized analytical frameworks, and clear evidence of clinical utility. Future advances are likely to arise from the integration of LB with multi-omics technologies, artificial intelligence-based analytical tools, imaging modalities, and clinicopathological parameters. In addition, insights derived from single-cell and spatial profiling approaches may improve our understanding of tumor heterogeneity and disease evolution. Rather than reflecting tumor burden alone, next-generation LB platforms may incorporate molecular signatures associated with specific tumor and microenvironmental cell states. Such integrated, biology-driven approaches could support the transition from isolated biomarker measurements toward comprehensive diagnostic frameworks, ultimately enabling more accurate, personalized, and clinically actionable strategies for early BC detection. These technologies may facilitate the identification of biologically relevant tumors and microenvironmental subpopulations that are most likely to contribute to the circulating biomarker pool.

## 11. Conclusions

LB has emerged as a minimally invasive approach for investigating BC, providing access to a broad spectrum of circulating biomarkers that reflect different aspects of tumor biology. CtDNA, CTCs, EVs, circulating RNAs, proteins, and other emerging analytes collectively offer unique opportunities for disease detection, monitoring, prognostic assessment, and evaluation of treatment response. Despite these advances, the application of LB in early-stage BC remains constrained by the limited release of tumor-derived material into the circulation, substantial biological heterogeneity, and persistent challenges related to assay standardization and analytical reproducibility.

Current evidence indicates that no single biomarker can adequately capture the complexity of early disease or consistently achieve the diagnostic performance required for routine clinical implementation. Consequently, future developments are expected to focus on integrated strategies that combine multiple classes of circulating biomarkers with complementary molecular information. The incorporation of multi-omics technologies, artificial intelligence-driven analyses, and insights derived from single-cell and spatial profiling may improve both biomarker discovery and the interpretation of circulating signals within their biological context.

Overall, LB represents one of the most promising developments in precision oncology for early BC. However, current evidence remains insufficient to support its routine implementation for population-based early detection. Future clinical adoption will depend on large prospective validation studies, standardized pre-analytical and analytical workflows, harmonized reporting criteria, and robust demonstration of clinical utility beyond existing imaging-based screening strategies. Rather than replacing conventional screening modalities, LB is more likely to complement current diagnostic approaches through integrated multi-analyte strategies. As these challenges are progressively addressed, LB has the potential to become an integral component of precision oncology, enabling more accurate, individualized, and biologically informed patient management.

## Figures and Tables

**Table 1:** Major extracellular vesicle-associated biomarkers and their clinical relevance in breast cancer. Overview of the principal extracellular vesicle (EV)-associated proteins, RNAs, lipids, and nucleic acid biomarkers investigated in breast cancer (BC), highlighting their reported associations with diagnosis, prognosis, metastasis, treatment response, and disease progression.

EV Biomarker Category	Specific Biomarkers	Association with Breast Cancer (BC)	Reference
**Proteins**	MUC1, HER2/ERBB2, CEA, Del-1, EpCAM, survivin-2B, MMP-1, EGFR, PSMA, VEGF, EPHA2, GRB7, EDIL3, UCH-L1, TrpC5, PD-L1, CD14, CA15-3, CD82, CD105, CA125, CD146, CD151, CD326,	- Elevated in BC derived EVs - May help distinguish metastatic vs. non-metastatic disease vs. healthy control - Some may be linked to subtype-specific biological pathways - EpCAM expression is higher in metastatic BC - PSMA is associated with disease progression - EDIL3 enhances BC progression - UCH-L1 & TrpC5 may be associated with chemotherapy response and drug resistance, respectively - PD-L1 is associated with immune evasion and may be used as prognostic marker	[132,133,134,136,138,139,148]
**RNAs**	(**miRNAs**) miR-10b, miR-21, miR-105, miR1246, miR-373, miR-375, miR-222, miR-155, miR-19a, miR-181b, miR-24, let-7a, miR-92b, miR-130a, miR-149, miR-200, miR-328, miR-423-5p, miR-602, miR-105, miR-122, miR-770, miR-17, miR-30a, miR-100, miR-222, miR-106b-5p, miR-18a-5p, miR421, miR128-1, miR128-2	- Higher in BC patients - Linked to BC development and early diagnosis - May be associated with increased invasion, endothelial disruption and metastasis - miR-21 linked with tumor size and circulating tumor cells - miR-105 potentially predictive of metastasis and prognosis - miR-373 associated with TNBC - miR-770 suppresses DOX resistance mechanism in TNBC cells	[127,132,134,142,148,149]
	(**lncRNAs**) H19, HOTAIR, SNHG14, lncRNA XIST	- Elevated in BC exosomes - HOTAIR and H19 are linked to aggressive disease & poor prognosis - SNHG14 is associated with trastuzumab resistance - lncRNA XIST is related to tumour recurrence	[132,134,148]
	(**mRNAs**) GRM1, S100A8, GRIK1, CSTA, H6PD, IGF2BP1, TPT1, MDM4 TK1, CDK9, GSTP1	- May assist with distinguishing BC vs. healthy controls - GSTP1 is associated with drug resistance - TK1 & CDK9 are associated with poor response to palbociclib (CDK inhibitors)	[132,134]
**Lipids**	Cer, SM, HexCer PC, PE, PI lysophospholipids	- Elevated in BC derived EVs - PS & HexCer associated with metastatic BC	[144]

**Table 2:** “Emerging circulating biomarkers for early breast cancer: clinical potential and current limitations”. Overview of major circulating RNA-, protein-, platelet-, and immune-related biomarkers investigated for early breast cancer (BC) detection and monitoring, highlighting their principal strengths and current limitations.

Free Serum/Plasma Circulating Biomarkers	Main Strength	Main Limitations
miRNAs	Widely studied, associated with early detection/prognosis/metastasis/treatment response, multi-miRNA panels show high diagnostic accuracy	Limited specificity, methodological variability, lack of clinically validated panels
lncRNAs	Potentially more disease-specific than miRNAs, useful for prognosis and subtype classification	Require larger validation studies before clinical implementation, lack of standardized detection methods
Proteins	Reflect tumor biology and immune responses, useful for prognosis and treatment monitoring, proteomic approaches may contribute to multi-marker panels	Low sensitivity and specificity for early BC detection, influenced by systemin inflammation and other non-cancer conditions, individual proteins usually perform poorly as standalone biomarkers
TEPs	Detect early-stage BC, provide useful information about tumor origin and molecular subtype, diagnostic performance appears independent of tumor stage, potentially powerful tool when combined with machine learning approaches	Highly sensitive to pre-analytical and technical factors (sample collection, processing, storage), challenging reproducibility, clinical utility remains unproven
AAbs	Detectable in early tumor development, stable in circulation, high potential for multi-antibody panels	Low sensitivity when used individually, limited large-scale clinical validation, require combination with other markers for improved accuracy

**Table 3:** “Clinical Utility and Current Limitations of Liquid Biopsy in Early Breast Cancer”. Overview of landmark studies evaluating liquid biopsy technologies in early breast cancer (BC). While circulating biomarkers identify molecular recurrence months before radiological progression and improve risk stratification, their integration into routine clinical practice is currently limited by low biomarker abundance in early-stage disease, insufficient standardization, and the absence of prospective interventional data demonstrating clinical benefit.

Clinical Trial	Patient Cohort	Liquid Biopsy Approach	Clinical Application	Key Findings & Clinical Limitations	Key Limitations
**CCGA Study** [193]	Individuals undergoing screening for localized stage I–III breast cancer (BC)	cfDNA methylation profiling using NGS-based MCED assay	Asymptomatic cancer screening	Demonstrated exceptionally high specificity (99.5%) and accurate tissue-of-origin prediction	Limited sensitivity for stage I disease due to low ctDNA abundance and minimal tumor shedding
**BRE12-158 Trial** [199]	Early Triple Negative BC (TNBC) patients with residual disease following neoadjuvant chemotherapy	Plasma ctDNA sequencing for tumor-specific somatic alterations	Post-treatment prognostic stratification	Persistent ctDNA detection was strongly associated with distant recurrence and inferior survival outcomes	Clinical benefit of therapy escalation based on ctDNA status has not yet been established
**Prospective Cohort** [196]	Early BC patients after definitive surgery and curative-intent treatment	Personalized longitudinal ctDNA monitoring	Minimal residual disease (MRD) detection	Molecular relapse was identified a median of 8.9 months before conventional imaging	Unclear whether earlier detection translates into improved survival or altered clinical outcomes
**Molecular Relapse** [197]	Early-stage BC patients during postoperative surveillance	Personalized digital PCR and targeted NGS assays	Molecular recurrence monitoring	Successfully detected subclinical recurrence and tracked disease evolution prior to overt relapse	Clinical utility for guiding treatment adaptation remains investigational

**Table 4:** Methodological profiles, analytical sensitivity thresholds, and clinical limitations of prominent commercial ctDNA assays.

Commercial Assay	Assay Type & Methodology	Sensitivity Threshold (Limit of Detection)	Primary Strengths & Clinical Utility	Key Technical & Clinical Weaknesses
Guardant360^®^/FoundationOne^®^ Liquid CDx [190,192]	Hybrid-capture NGS	~0.1–0.5% VAF	Provides comprehensive genomic profiling (CGP) across multiple genes	Low sensitivity in early-stage disease due to low tumor shedding of ctDNA into the circulation CHIP false-positive (requires WBC sequencing to filter)
Signatera™ (Natera) [196,199]	Tumor-informed, patient-specific multiplex PCR (mPCR) + NGS	~0.01% VAF (Ultra-sensitive)	Gold standard for MRD tracking Detects molecular recurrence months before imaging	Prolonged turnaround time (requires tissue biopsy first) Blind to mutations outside the custom panel
ddPCR Platforms (e.g., Bio-Rad) [197]	Digital Droplet PCR (ddPCR) target amplification	~0.01–0.1% VAF	Cost-effective with rapid turnaround time Ideal of longitudinal tracking pf known hotspot mutations	Severe multiplexing limits (tracks 1–4 targets) Not able to discover novel variants
MCED Assays (e.g., Galleri^®^/CancerSEEK) [193,198]	Multi-Cancer Early Detection (MCED) via cfDNA methylation or multi-analyte profiling	Variable threshold (dependent on genome-wide features)	High specificity (99.5%) and accurate tissue-of-origin prediction	Suboptimal sensitivity in localized Stage I disease

**Table 5:** “Major Technological Challenges and Emerging Solutions for Liquid Biopsy in Early Breast Cancer”. Overview of the principal technological barriers limiting the clinical implementation of liquid biopsy (LB) in early breast cancer (BC). The table summarizes the biological and technical origins of these challenges, their impact on diagnostic performance and clinical translation, and emerging strategies aimed at improving sensitivity, specificity, reproducibility, and biological resolution.

Technological Challenge	Biological/Technical Basis	Clinical Consequence	Emerging Solutions
Low biomarker abundance	Minimal tumor burden and limited biomarker shedding in early-stage disease	Reduced sensitivity and increased false-negative rates	Ultra-deep sequencing, multi-analyte panels, longitudinal monitoring
Sensitivity–specificity trade-off	Detection of rare tumor-derived signals within a large background of normal cfDNA	Increased false-positive and false-negative results	Error-corrected sequencing, integrated biomarker approaches
Clonal hematopoiesis (CHIP)	Age-related somatic mutations in hematopoietic cells	Misclassification of non-tumor variants as tumor-derived alterations	Matched leukocyte sequencing and improved bioinformatic filtering
Pre-analytical variability	Differences in blood collection, processing, storage, and sample handling	Poor reproducibility across laboratories	Standardized protocols and international quality-control guidelines
Technical noise	PCR artifacts, sequencing errors, and sample contamination	Reduced analytical accuracy	Error-suppression algorithms and optimized sequencing workflows
Inter-platform heterogeneity	Diverse sequencing technologies and proprietary bioinformatic pipelines	Limited cross-study comparability and validation	Harmonized analytical frameworks and reference standards
AI and machine-learning limitations	Data scarcity, overfitting, and limited explainability	Restricted clinical translation and generalizability	Large multicenter datasets, external validation, explainable AI models
Intratumoral heterogeneity	Spatial and temporal diversity of tumor cell populations	Incomplete representation of disease biology	Spatial biology, single-cell profiling, and dynamic biomarker tracking
Clinical implementation barriers	High cost, infrastructure requirements, and computational demands	Limited accessibility in routine clinical practice	Scalable workflows and cost-effective multiplex technologies

**Table 6:** Comparative evaluation of major liquid biopsy (LB) biomarkers for early breast cancer (BC). Summary of their biological targets, principal strengths, limitations, representative diagnostic performance, and current clinical readiness.

Biomarker	Main Biological Information	Principal Strengths	Major Limitations	Current Clinical Applications	Clinical Readiness for Early BC
ctDNA	Mutations, methylation, fragmentomics	High specificity, MRD, actionable mutations	Low abundance in early BC, CHIP	Molecular profiling, MRD detection, treatment monitoring	Established for molecular profiling/MRD, investigation for screening
CTCs	Intact tumor cells	Functional & phenotypic characterization	Extreme rarity, EMT	Prognosis (metastatic BC)	Limited for early detection
EVs	DNA, RNA, proteins	Stable cargo, reflects TME	Isolation not standardized	Early detection, disease monitoring (investigational)	Investigational
Circulating RNAs	miRNAs, lncRNAs, mRNAs	Easy detection, multiplexing	Limited specificity	Experimental	Investigational
Protein biomarkers	Secreted proteins	Clinically accessible assays	Poor early-stage sensitivity	Limited adjunctive use	Limited
Multi-analyte assays	Combined biomarkers	Highest diagnostic potential	Cost, standardization	Emerging	Most promising

## Data Availability

No new data were created or analyzed in this study. Data sharing is not applicable to this article.

## Abbreviations

The following abbreviations are used in this manuscript:

ADM	Automated Digital Microscopy
AI	Artificial Intelligence
ASCO	American Society of Clinical Oncology
BC	Breast Cancer
CAM	Collagen Adhesion Matrix
cdPCR	Chip-based Digital Polymerase Chain Reaction
CEA	Carcinoembryonic Antigen
cfDNA	Cell-Free DNA
CHIP	Clonal Hematopoiesis of Indeterminate Potential
CSCs	Cancer Stem Cells
CTC-chip	Circulating Tumor Cell Chip
CTCs	Circulating Tumor Cells
ctDNA	Circulating Tumor DNA
DCIS	Ductal Carcinoma In Situ
ddPCR	Droplet Digital Polymerase Chain Reaction
DEP-FFF	Dielectrophoretic Field-Flow Fractionation
DFS	Disease-Free Survival
dPCR	Digital Polymerase Chain Reaction
DTCs	Disseminated Tumor Cells
DOX	Doxorubicin
EGFR	Epidermal Growth Factor Receptor
EMT	Epithelial-to-Mesenchymal Transition
EpCAM	Epithelial Cell Adhesion Molecule
EPISPOT	Epithelial Immunospot Assay
ESR1	Estrogen Receptor 1
EVs	Extracellular Vesicles
FAST	Fiber-Optic Array Scanning Technology
FDA	Food and Drug Administration
HER2	Human Epidermal Growth Factor Receptor 2
HR+	Hormone Receptor-Positive
ICC	Immunocytochemistry
ISET	Isolation by Size of Epithelial Tumor Cells
LB	Liquid Biopsy
lncRNA	Long Non-Coding RNA
MACS	Magnetic-Activated Cell Sorting
MCED	Multi-Cancer Early Detection
MeDIP-Seq	Methylated DNA Immunoprecipitation Sequencing
MEMS	Microelectromechanical Systems
miRNA	MicroRNA
MRD	Minimal Residual Disease
MSCs	Mesenchymal Stem Cells
MVs	Microvesicles
NGS	Next-Generation Sequencing
PCR	Polymerase Chain Reaction
PIK3CA	Phosphatidylinositol-4,5-Bisphosphate 3-Kinase Catalytic Subunit Alpha
RNA	Ribonucleic Acid
TEPs	Tumor-Educated Platelets
TME	Tumor Microenvironment
TNBC	Triple-Negative Breast Cancer
VEGF	Vascular Endothelial Growth Factor
